# A randomized trial of I‐SLEEP: A patient education and empowerment intervention on inpatient sleep duration and medical sleep disruptions

**DOI:** 10.1002/jhm.70273

**Published:** 2026-02-06

**Authors:** Aashna Sunderrajan, John Cursio, Noah Mason, Maxx Byron, Maylyn Martinez, Nicola Orlov, Kristen L. Knutson, Babak Mokhlesi, Valerie G. Press, David O. Meltzer, Vineet M. Arora

**Affiliations:** ^1^ Department of Medicine University of Chicago Chicago Illinois USA; ^2^ Department of Public Health Sciences University of Chicago Chicago Illinois USA; ^3^ Department of Pediatrics University of Colorado Boulder Colorado USA; ^4^ Chicago School of Professional Psychology Chicago Illinois USA; ^5^ Department of Pediatrics University of Chicago Chicago Illinois USA; ^6^ Center for Circadian and Sleep Medicine Northwestern University Evanston Illinois USA; ^7^ Department of Medicine Rush University Chicago Illinois USA

## Abstract

**Background:**

Sleep is essential for recovery in hospitalized patients, yet frequent disruptions from medical care make rest difficult. Most prior efforts have focused on environmental modifications, often overlooking patients' role in advocating for their sleep.

**Objectives:**

This study evaluated the effectiveness of the Inpatient Sleep Loss: Educating and Empowering Patients (I‐SLEEP) intervention in improving sleep duration and reducing medical care disruptions among hospitalized patients.

**Methods:**

In this single‐center randomized controlled trial (NCT04151251), general medicine patients at the University of Chicago were randomized to either I‐SLEEP (sleep education, advocacy questions, and a sleep kit) or standard care (sleep kit alone) between July 2019 and March 2023. The primary outcome was patient‐reported sleep duration. Secondary outcomes included actigraphy‐measured sleep duration and continuity (efficiency and wake after sleep onset), as well as patient‐reported sleep disruptions. Mixed effects models adjusted for covariates were used for analysis.

**Results:**

A total of 194 participants were enrolled. There were no significant differences in demographic characteristics between groups. Sleep duration and continuity did not differ significantly between groups. However, patients receiving I‐SLEEP reported fewer disruptions from vital sign monitoring (63% vs. 75%, *p* = .004), medication administration (49% vs. 61%, *p* = .003), and laboratory draws (57% vs. 68%, *p* = .009). These findings remained significant after adjusting for covariates.

**Conclusions:**

I‐SLEEP did not increase sleep duration or continuity but reduced medical care disruptions. These findings suggest that patient education and empowerment may be effective strategies for reducing preventable care‐related sleep disruptions in hospitals. Further research should examine implementation at scale and potential long‐term benefits.

## Visual Abstract

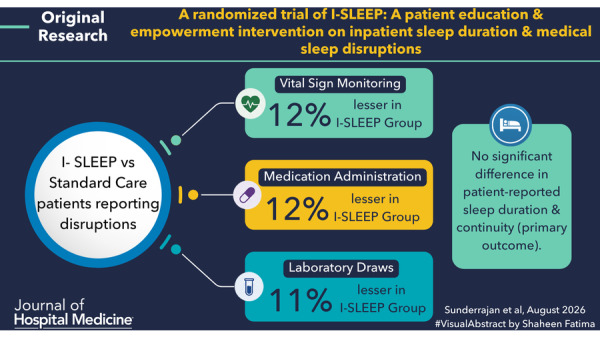

## INTRODUCTION

Sleep is fundamental to healing and recovery, yet hospitalized patients often face significant challenges in obtaining adequate rest.^1^ The hospital environment presents multiple barriers to sleep, including persistent noise from alarms, staff conversations, hallway traffic, and medical equipment,^2^, ^3^, ^4^ as well as lighting that remains on or is frequently turned on at night.^2^, ^3^, ^5^ Beyond these ambient factors, sleep is also disrupted by overnight medical care activities, including vital sign monitoring, medication administration, and laboratory draws.^6^

The consequences of inpatient sleep loss are wide‐ranging. Poor sleep during hospitalization is associated with complications such as delirium,^7^ immune suppression,^8^ and cardiometabolic dysfunction.^9^, ^10^ Patients who experience poor sleep during hospitalization are also more likely to report fatigue,^11^ low energy,^12^ and reduced quality of life in the weeks following discharge,^13^, ^14^ and they face higher risks of hospital readmission within 30 days^15^ and lower survival rates at 1 year.^16^

Interventions to improve inpatient sleep have historically included environmental modifications, such as sensory masking with sleep aids^17^, ^18^ or changes in staff routines.^6^, ^19^, ^20^ While sleep aids, such as eye masks and earplugs, have shown to help patients fall asleep more easily,^21^ stay asleep longer,^22^ and experience fewer awakenings,^23^ their real‐world effectiveness varies due to patient discomfort, intolerance, or disorientation.^24^, ^25^ Similarly, although staff‐focused initiatives have shown to meaningfully decrease sleep disruptions and improve patient satisfaction,^26^ their implementation can be difficult to sustain in the context of staffing constraints and burnout.^27^ As might be expected, fewer than half of the top‐ranked hospitals report satisfaction with their strategies for improving inpatient sleep.^28^

By comparison, far less attention has been directed toward the role patients themselves can play in protecting their sleep. Broadly, a growing body of research demonstrates that education and empowerment can meaningfully improve engagement and outcomes across clinical contexts. Studies of hospitalized and ambulatory patients have shown that higher patient activation is associated with fewer unmet medical needs, greater adherence to treatment recommendations, improved communication with clinicians, and better health outcomes overall.^29^, ^30^ In the context of sleep, education‐ and empowerment‐based approaches have similarly been associated with improvements in sleep outcomes across diverse populations.^31^, ^32^, ^33^ In the hospital setting, greater perceived control over the sleep environment has been associated with longer sleep duration, better quality, and fewer noise‐related disruptions.^34^ These findings suggest that actively involving patients in decision‐making could complement traditional environmental approaches and address aspects of sleep that system‐level changes alone may not resolve.

Despite this potential, no prior study has evaluated whether educating and empowering hospitalized patients can increase inpatient sleep duration and reduce medical care disruptions. To address this gap, we developed the Inpatient Sleep Loss: Educating and Empowering Patients (I‐SLEEP) intervention. I‐SLEEP combines targeted sleep hygiene education with strategies to empower patients to work with their care team to minimize preventable medical care disruptions. In a single‐arm pilot study, I‐SLEEP was well‐received and associated with reduced patient‐reported sleep disruptions and greater perceived control over the sleep environment,^35^ supporting its feasibility for broader evaluation.

The present randomized controlled trial is designed to evaluate the effectiveness of I‐SLEEP in a general medicine inpatient population. We aim to determine whether patients receiving the intervention will experience longer sleep duration and fewer medical care disruptions compared with those receiving standard care.

## METHODS

This was a single‐center randomized controlled trial of I‐SLEEP, with hospitalized patients recruited from the University of Chicago between July 2019 and March 2023. This study was registered on ClinicalTrials.gov under trial number NCT04151251 before participant enrollment (https://clinicaltrials.gov/study/NCT04151251?a=9) and was approved by the University of Chicago Institutional Review Board (protocol number 19‐0169). This study adhered to the Consolidated Standards of Reporting Trials (CONSORT) guidelines for reporting randomized controlled trials. A CONSORT checklist is provided with the Supporting Information.

### Participants

Adult general medicine patients at the University of Chicago were enrolled in this study. Participants were excluded from this study if they met one or more of the following criteria: (1) were admitted to the hospital longer than 72 h ago, (2) had a preexisting sleep disorder (i.e., obstructive sleep apnea, narcolepsy), (3) were transferred from the intensive care unit (ICU) or another hospital, (4) had noncontact (i.e., droplet, strict, or airborne) isolation precautions, (5) were unable to walk or were on strict bedrest, (6) had cognitive or sensory deficits precluding informed consent, or (7) were readmitted to the hospital within 2 weeks of their last discharge. Participant exclusion criteria were selected to avoid conditions that could interfere with actigraphy‐based sleep measurement, limit the focus to acute sleep loss during the current hospitalization, and ensure participants could participate in the study.^14^ All participants provided written informed consent.

### Intervention protocol

I‐SLEEP was developed through an iterative process that incorporated input from both patients and clinical staff. Before trial implementation, we conducted a needs assessment with hospitalized patients and nursing staff to identify the most prevalent sources of sleep disruption. Medical care disruptions, including overnight vital sign monitoring, medication administration, and laboratory draws, were consistently identified as the most common and preventable causes of disturbed sleep, followed by environmental factors such as light and noise.^2^, ^6^, ^35^

**Figure 1:**
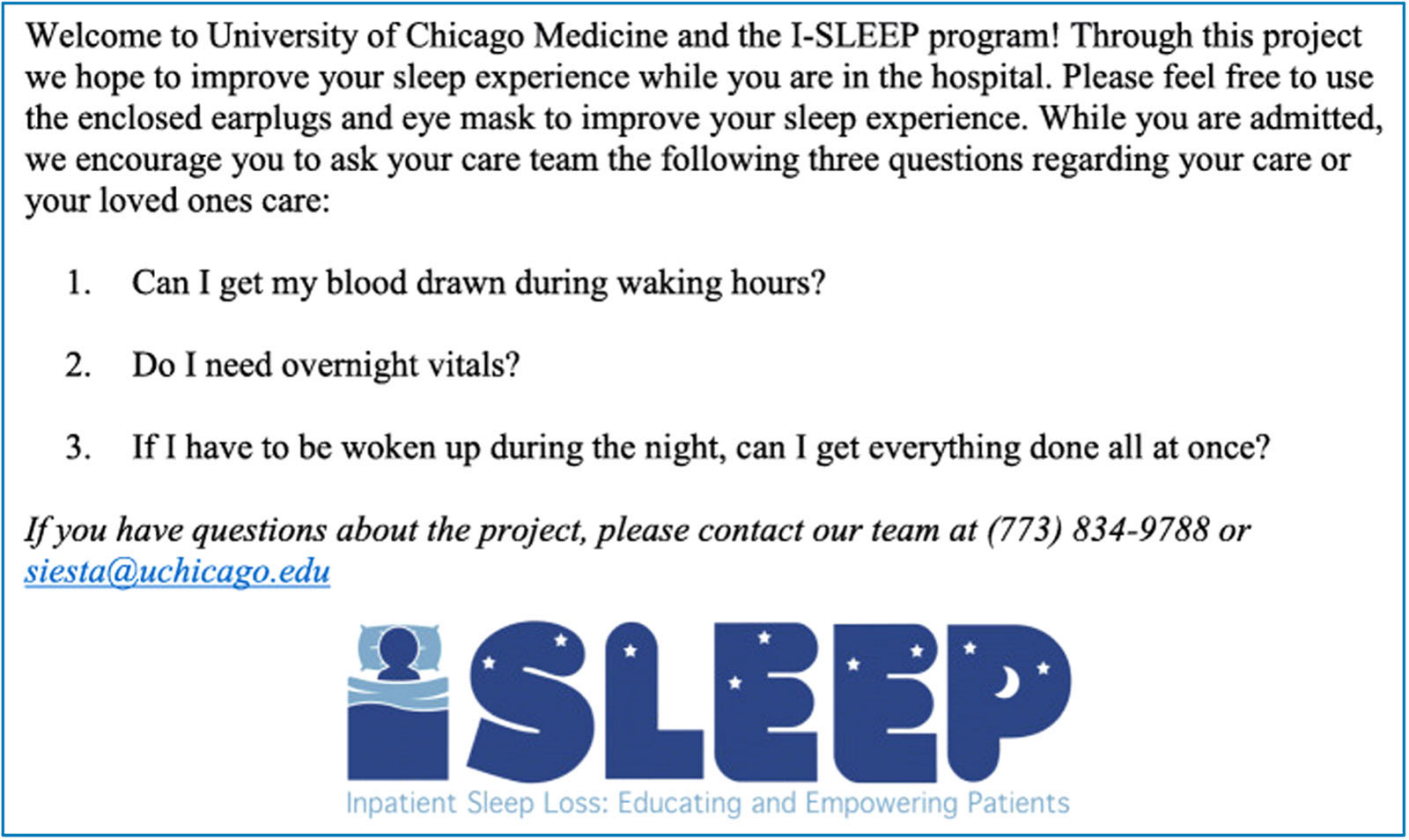
Notecard provided to participants in the Inpatient Sleep Loss: Educating and Empowering Patients (I‐SLEEP) group. The notecard listed three questions that participants were encouraged to ask their care team throughout hospitalization to help minimize nighttime disruptions.

I‐SLEEP consisted of three integrated components designed to educate and empower patients to protect their sleep during hospitalization. At the bedside, participants viewed a 5‐min educational video on an iPad that highlighted the importance of sleep, described common hospital‐based disruptions, and provided practical strategies to improve rest—including the use of nonpharmacologic sleep aids, such as eye masks and earplugs (see Supporting Information). Participants also received a brochure, adapted from the National Heart, Lung, and Blood Institute's (NHLBI) Tips to Get Healthy Sleep,^36^ which reinforced key messages from the video and served as a cognitive prompt to support sleep advocacy. Finally, participants were given a sleep kit containing an eye mask, earplugs, and a notecard with three suggested questions to ask their care team to help minimize preventable nighttime disruptions: (1) Can I get my blood drawn during waking hours? (2) Do I need overnight vitals? and (3) If I have to be woken up during the night, can I get everything done all at once? (See Figure 1). To ensure the materials were patient‐centered and accessible, hospitalized patients reviewed the video script and brochure, leading to refinements for clarity, cultural relevance, and usability.

Participants in standard care received the NHLBI brochure^36^ and a sleep kit containing only the eye mask and earplugs, without additional education or guidance to engage their care team. We selected the NHLBI brochure because it is a publicly available, evidence‐based patient education resource that offers general sleep hygiene guidance appropriate for routine care. Including the brochure and a basic sleep kit in both arms standardized access to foundational sleep information and materials, allowing the trial to isolate the added effect of the I‐SLEEP education and empowerment components.

All intervention and control materials were delivered in person by study staff. Participants in both groups continued to receive usual medical care as determined by their clinical team, including all standard diagnostics, treatments, and care routines unrelated to the study.

### Outcomes

Data were collected before the intervention and then daily throughout the hospitalization period to capture both baseline and evolving sleep patterns. The prespecified primary outcome for this study was patient‐reported sleep duration. Sleep duration was selected as the primary outcome because it represents a clinically meaningful and easily interpretable indicator of sleep restoration, integrating the cumulative effects of sleep fragmentation, efficiency, and continuity. Patient‐reported sleep duration was assessed using the Karolinska Sleep Log (KSL), which is a validated self‐report instrument that captures multiple dimensions of sleep, including onset, duration, and quality.^37^ For this analysis, we used the KSL item asking participants to report their total sleep time from the previous night (in minutes).

Secondary outcomes included actigraphy‐based measures of sleep—specifically sleep duration, sleep efficiency, and wake after sleep onset (WASO)—as well as patient‐reported sleep disruptions. Sleep efficiency and WASO were included as indicators of sleep continuity, reflecting the degree of sleep fragmentation and restfulness throughout the night. Actigraphy was selected to complement self‐reported measures and provide an objective, continuous assessment of sleep duration and continuity in the hospital environment. Actigraphy‐measured sleep was measured noninvasively using wrist actigraphy (Actiwatch Spectrum, Philips, Amsterdam, Netherlands) worn on the nondominant wrist. Actiwatches record movement via accelerometry, with each movement generating a variable voltage that is processed digitally, sampled at 32 Hz, and integrated over a user‐defined epoch.^38^ For this analysis, we focused on nocturnal sleep periods, with bedtimes and wake times identified from the KSL. Our team has previously established concurrent validity of actigraphy‐measured sleep in hospitalized patients against other sleep measures (e.g., Karolinska Sleep Quality Index) and demonstrated discriminant validity relative to hospital noise levels.^4^

Patient‐reported sleep disruptions were included to capture the specific, care‐related interruptions most directly targeted by the intervention, providing a mechanistic link between patient experience and observed sleep outcomes. This was measured using the Modified Potential Hospital Sleep Disruptions and Noises Questionnaire (PHSDNQ), an adaptation of the Freedman et al.^39^ tool developed by our team. The PHSDNQ includes 10 items assessing disruptions during the prior night, each rated on a 5‐point Likert‐type scale (1 = “Minimal disruption” to 5 = “Extreme disruption”). Items cover medical interventions (vitals, medications, labs), symptoms (pain, anxiety), and environmental factors (noise, alarms, temperature). For this analysis, we focused on medical disruptions from vital sign monitoring, medication administration, and laboratory draws. Consistent with prior work, scores ≥ 2 were classified as “sleep disruption.”^2^ This measure has been validated against actigraphy.^2^

To evaluate participants' satisfaction with and uptake of I‐SLEEP, we administered a brief questionnaire. Satisfaction with the educational video and brochure were each rated on a 5‐point Likert‐type scale (1 = “Not at all satisfied” to 5 = “Extremely satisfied”), while uptake was assessed by asking whether participants had posed each of the three recommended questions to their care team. For any question not asked, participants were prompted to indicate the reason for not doing so, such as not remembering, perceiving it as inapplicable, feeling uncomfortable, or lacking the opportunity to do so.

Enrollment required medical clearance from the treating clinical team. As an additional safeguard, we monitored unplanned ICU transfers and rapid response activations resulting from reduced overnight monitoring (e.g., deferred vital sign checks) as balancing measures to detect unintended clinical consequences. Nursing staff were also periodically queried to identify any concerns or adverse events related to fewer nighttime disruptions.

### Data analysis

Study data were collected and managed in REDCap.^40^ All randomized participants with available outcome data were analyzed according to the intention‐to‐treat principle. The effects of I‐SLEEP on sleep duration, sleep continuity, and sleep disruptions were evaluated using mixed‐effects models, adjusting for study day, age, gender, race, ethnicity, body mass index, apnea risk, and Charlson Comorbidity Index, with a random intercept for each participant. To assess changes over time, models included an interaction term between study day and intervention group, enabling evaluation of intervention effects as participants approached discharge. Missing data were addressed using listwise deletion, as mixed‐effects models can accommodate some missingness under the assumption of data missing at random. Statistical significance was set at *p* < 0.05.

### Randomization procedure

Participants were randomly assigned to either I‐SLEEP or standard care using a permuted‐block randomization scheme with variable block sizes to ensure balanced allocation and minimize predictability. The study statistician (J.C.) generated the random sequence using statistical software, incorporating a random seed to allow for reproducibility. Treatment assignments were labeled as “1” and “2” and were securely uploaded into REDCap, hosted on a password‐protected University of Chicago server.

Allocation concealment was maintained by restricting access to the randomization list; only the study statistician had access to the sequence and unblinded allocation list. Because the intervention involved behavioral education, blinding of patients and clinical staff was not feasible. However, outcome assessors were blinded to group assignments throughout hospitalization to minimize bias in data collection.

### Trial design

This single‐center, parallel‐group randomized controlled trial used a 1:1 allocation ratio to assign participants to either I‐SLEEP or standard care for the duration of the study. Equal allocation allowed balanced comparisons between groups. The trial employed a superiority framework to evaluate whether I‐SLEEP produced better outcomes—specifically, longer sleep duration and sleep continuity, and fewer patient‐reported sleep disruptions—than standard care, providing a rigorous evaluation of the intervention's effectiveness in a real‐world inpatient setting.

### Sample size

Based on our prior work,^34^ we estimated that participants in standard care would obtain a mean of 333 min of sleep (SD = 182 min). Using an alpha level of 0.05, a total sample size of 256 participants (128 per group) was calculated to provide 80% power to detect a between‐group difference of 45 min in sleep duration.

### Futility analysis

As the study neared the planned conclusion of its NIH funding period, enrollment remained below target, in part due to recruitment disruptions caused by the coronavirus disease 2019 (COVID‐19) pandemic earlier in the study period. At that stage, only 194 participants had been enrolled, with 96 participants assigned to I‐SLEEP and 98 to standard care—fewer than the a priori sample size target. A futility analysis was therefore conducted to evaluate whether continued enrollment was likely to produce a statistically significant result. Based on the observed mean and standard deviation of sleep duration, the futility statistic was calculated as 0.99, indicating an extremely high probability that additional enrollment would not change the study's findings. The decision to terminate enrollment was reviewed and approved by both the study statistician and the Data and Safety Monitoring Board, and the trial was closed to further recruitment. Apart from this early termination based on the preplanned futility analysis, no substantive protocol modifications occurred after trial initiation.

## RESULTS

**Figure 2:**
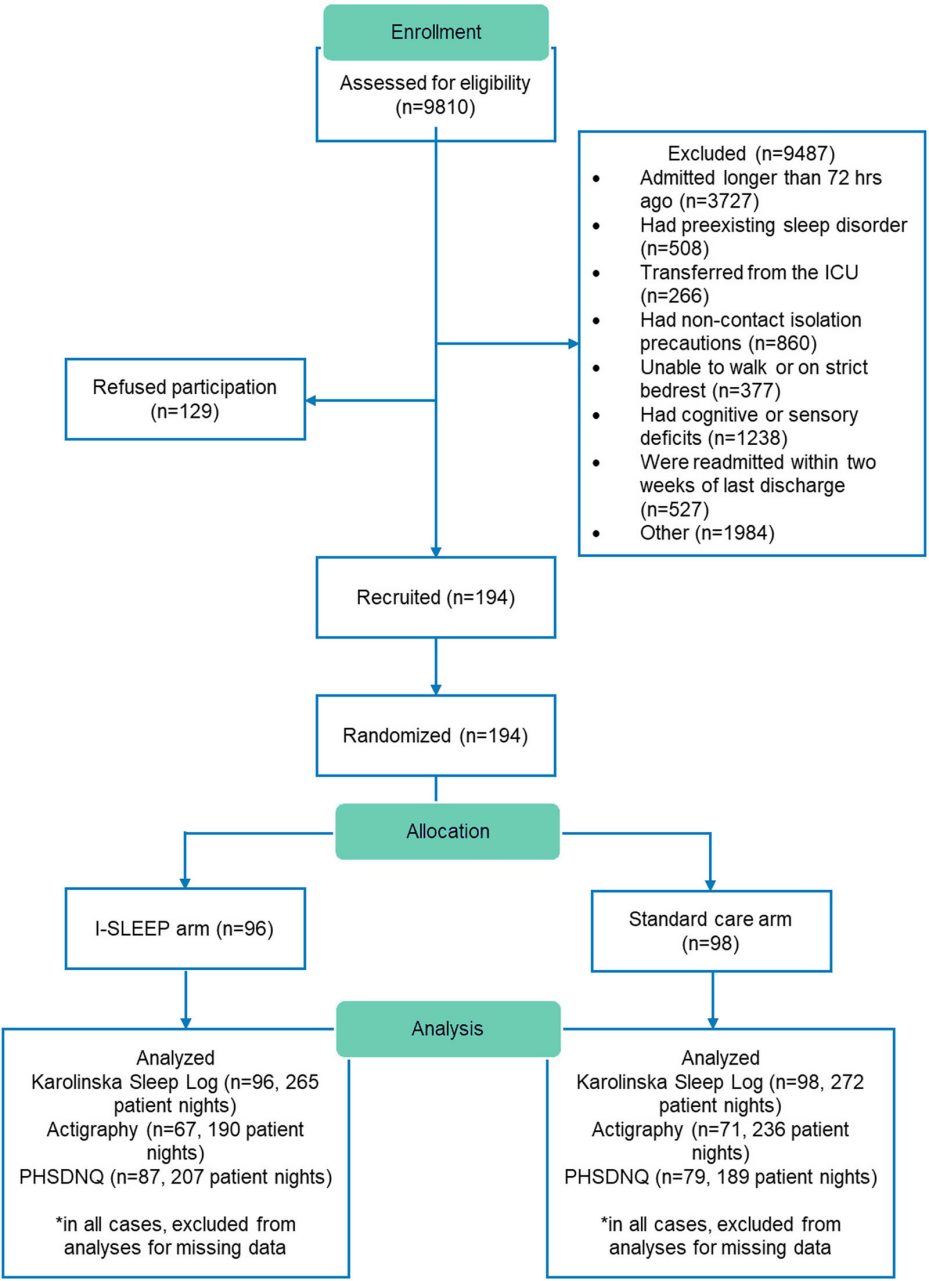
CONSORT flow diagram of participant enrollment, allocation, and analyses. The diagram illustrates the number of individuals assessed for eligibility, randomized to each intervention group, and included in the final analysis. Reasons for exclusions or withdrawals are also detailed. CONSORT, Consolidated Standards of Reporting Trials; I‐SLEEP, Inpatient Sleep Loss: Educating and Empowering Patients.

The study sample consisted of 194 participants (See Figure 2). The mean age of participants in the study was 53.4 years (SD = 16.9). Most participants identified as female (63%), Black (89%), and non‐Hispanic (95%). There were no statistically significant differences between groups in baseline demographic or clinical characteristics, including age, gender distribution, racial and ethnic composition, body mass index, or comorbidity burden (see Table 1). No adverse events or unintended effects were reported in either group during the study.

**Table 1:** Participant demographics.

Variable	Total, *N* = 194	I‐SLEEP, *N* = 96	Standard care, *N* = 98	*p* value
Age M (SD)	53.4 (16.9)	52.6 (17.1)	54.2 (16.8)	.51
Female *n* (%)	121 (63.0)	64 (66.7)	57 (59.4)	.29
Race *n* (%)				.14
African American	170 (89.0)	82 (87.2)	88 (90.7)	
White	13 (6.8)	8 (8.5)	5 (5.2)	
More Than One	5 (2.6)	1 (1.1)	4 (4.1)	
Unknown	3 (1.6)	3 (3.2)	0 (0.0)	
Hispanic *n* (%)				.08
Yes	6 (3.1)	2 (2.1)	4 (4.1)	
No	181 (94.8)	88 (93.6)	93 (95.9)	
Unknown	4 (2.1)	4 (4.3)	0 (0.0)	
BMI M (SD)	28.8 (9.1)	29.4 (8.7)	28.1 (9.5)	.35
Prior hospitalization *n* (%)	139 (80.4)	69 (80.2)	70 (80.5)	.97
COPD or asthma *n* (%)	49 (25.3)	26 (27.1)	23 (23.4)	.56
Diabetes *n* (%)	41 (21.1)	26 (27.1)	15 (15.3)	.04
Congestive heart failure *n* (%)	7 (3.6)	5 (5.2)	2 (2.0)	.28
End‐stage renal disease *n* (%)	16 (8.3)	7 (7.3)	9 (9.2)	.63

Abbreviations: BMI, body mass index; COPD, chronic obstructive pulmonary disease; I‐SLEEP, Inpatient Sleep Loss: Educating and Empowering Patients.

Patient‐reported sleep duration, measured using the KSL, was modestly higher in I‐SLEEP compared with standard care (430 vs. 416 min), though the difference was not statistically significant (*p* = .80). Actigraphy‐measured sleep duration similarly did not differ significantly between groups (333 vs. 330 min, *p* = .87). In secondary analysis examining the interaction between study day and intervention group, participants receiving I‐SLEEP demonstrated an average increase of 6.6 min of sleep per night (SE = 6.1 min) for each additional day of hospitalization; however, this trend did not reach statistical significance (*p* = .28). Actigraphy‐derived measures of sleep continuity similarly showed no statistically significant differences between groups. Sleep efficiency was comparable in I‐SLEEP and standard care (68% vs. 67%, *p* = .78), as was WASO (63 vs. 58 min, *p* = .35).

**Figure 3:**
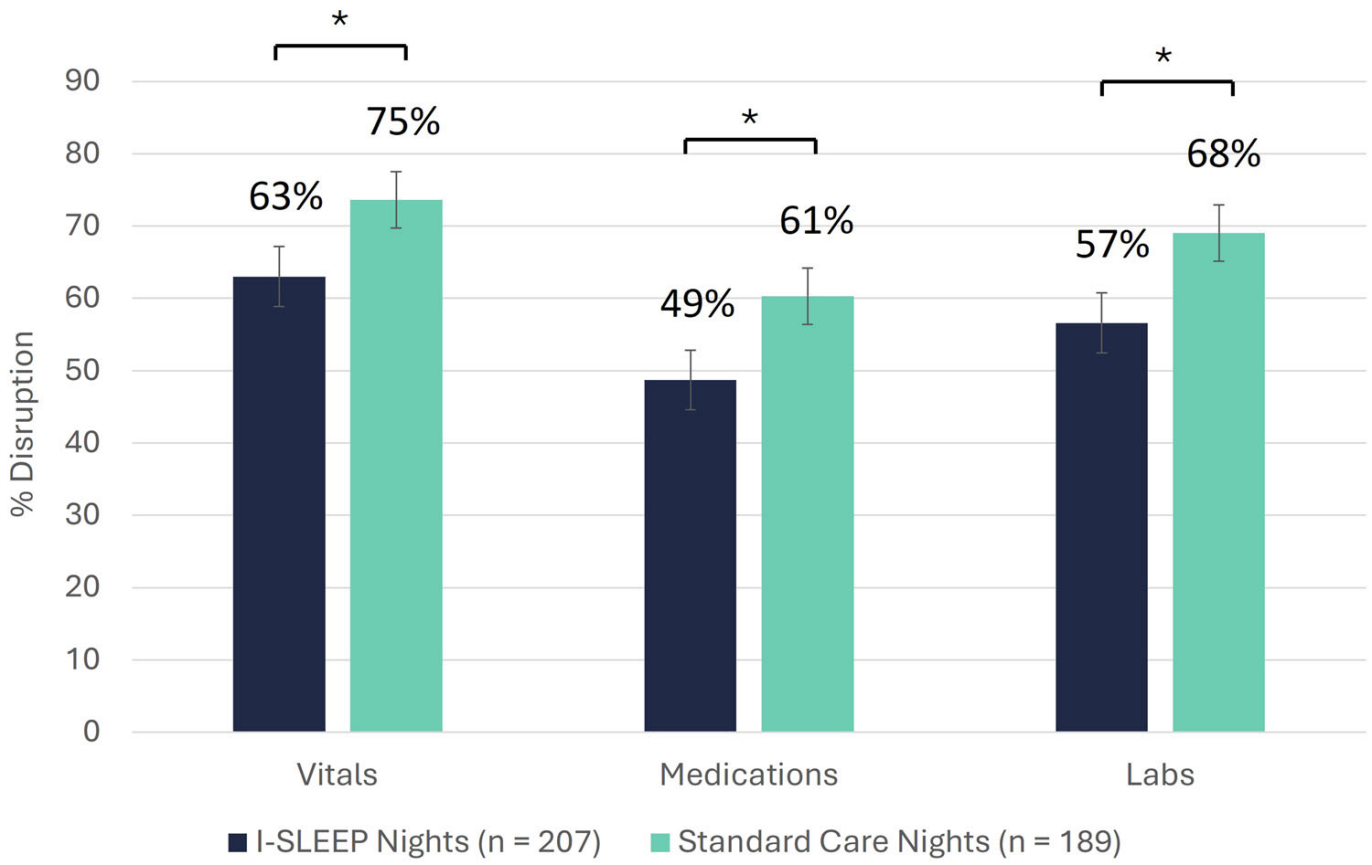
Percentage of participants reporting sleep disruptions due to vital sign monitoring, medication administration, and laboratory draws, by study group. Participants in I‐SLEEP reported significantly fewer disruptions across all three categories compared with those in standard care (*p* < .01 for all comparisons). I‐SLEEP, Inpatient Sleep Loss: Educating and Empowering Patients.

In contrast, participants in I‐SLEEP reported significantly fewer sleep disruptions related to vital sign monitoring (63% vs. 75%, *p* = .004), medication administration (49% vs. 61%, *p* = .003), and laboratory draws (57% vs. 68%, *p* = .009) compared with those in standard care (See Figure 3). These differences remained statistically significant after adjusting for covariates.

Satisfaction with I‐SLEEP was high, with 94.7% of participants satisfied with the brochure and 96.8% satisfied with the video. Although most participants (83.2%) reported feeling empowered to ask their care team to reduce medical care disruptions, actual uptake of the intervention questions was lower, with only 40% asking at least one suggested question. The most common reasons for not asking any questions were not having the opportunity (32.3%), not feeling comfortable (32.3%), and not remembering (27.3%) to ask.

## DISCUSSION

This randomized controlled trial evaluated the effectiveness of I‐SLEEP—an intervention designed to educate and empower hospitalized patients—on inpatient sleep duration and disruptions. While the intervention did not significantly increase sleep duration or improve indices of sleep continuity, it was associated with fewer patient‐reported disruptions from vital sign monitoring, medication administration, and laboratory draws. These findings suggest that patient‐centered strategies may influence specific modifiable contributors to hospital sleep loss, even if their impact on overall sleep parameters are more limited.

Participants who received I‐SLEEP reported fewer perceived disruptions due to medical care, specifically vital sign monitoring, medication administration, and laboratory draws. These findings align with prior research identifying these same activities as common contributors to inpatient sleep loss^2^, ^6^, ^33^ and demonstrating that reducing them can improve perceived sleep quality.^6^, ^19^, ^20^, ^26^ By focusing on patient education and empowerment, rather than relying solely on environmental modifications, I‐SLEEP demonstrates that patients can play an active role in minimizing modifiable disruptions, potentially enhancing their overall hospital experience.

Despite these improvements, we observed no significant differences in sleep duration or sleep continuity. This pattern is consistent with prior work showing that even effective environmental or workflow changes rarely produce large, sustained gains.^41^ This likely reflects the multifaceted nature of hospital sleep, in which patients face multiple barriers to rest—including underlying illness, pain, and anxiety^2^, ^3^—that education and advocacy alone may not overcome. Therefore, reductions in perceived disruptions may not translate to measurable gains in duration or continuity without concurrent system‐level changes. Future work should test whether combining patient‐centered strategies like I‐SLEEP with other targeted efforts or supportive approaches that address stress, anxiety, and discomfort during hospitalization can yield larger and more sustained improvements in sleep.

Although this difference was not statistically significant, the pattern in which patient‐reported sleep duration exceeded actigraphy‐measured duration is noteworthy and aligns with prior research showing that subjective sleep duration often overestimates objective sleep duration.^42^, ^43^ This divergence likely reflects both perceptual and methodological differences—actigraphy may underestimate sleep by misclassifying quiet wakefulness as wake, while patients may consolidate fragmented rest or intermittent dozing into a single estimate of sleep. These findings underscore the importance of integrating both objective and subjective measures when evaluating inpatient sleep interventions, as each provides complementary insight into patients' sleep experiences.

Participant feedback underscores both the promise and the limitations of a patient empowerment approach. Satisfaction with I‐SLEEP was high, with most participants rating materials favorably and feeling empowered to request fewer nighttime disruptions. However, fewer than half asked at least one of the recommended questions, citing barriers such as lack of opportunity, discomfort initiating the conversation, and difficulty remembering the questions. These findings highlight that, while education can increase perceived agency, additional strategies—such as prompts or staff reinforcement—may be needed to translate intentions into action.

Other important limitations warrant consideration. This study was conducted at a single academic medical center, which may limit the generalizability of findings to other hospital settings. Recruitment was disrupted by the COVID‐19 pandemic, limiting our ability to reach the target sample size and potentially reducing statistical power to detect small but meaningful effects. Patients with preexisting sleep disorders, such as obstructive sleep apnea or narcolepsy, were excluded from participation, which may limit the applicability of findings to these higher risk populations. Additionally, because our disruption outcomes were based solely on patient reports, we cannot determine whether the observed differences reflect true reductions in overnight care activity or changes only in patient perception. These limitations underscore the need for replication in larger, multisite studies with diverse patient populations and using both patient‐reported and objective measures to more comprehensively evaluate the effects of I‐SLEEP.

The implications of our findings are significant for hospital‐based sleep promotion. Educating patients and equipping them with tools to advocate for their sleep needs may help mitigate preventable disruptions and improve the overall inpatient experience. Additionally, while total sleep did not change, fewer perceived disruptions may still yield benefits such as greater satisfaction, reduced stress, and improved recovery. Further research should explore whether empowering patients to manage their sleep could contribute to long‐term improvements in sleep hygiene and post‐discharge health outcomes.

In conclusion, while I‐SLEEP did not significantly extend sleep duration or sleep continuity, it reduced perceived medical care disruptions, suggesting value in incorporating patient engagement strategies into hospital sleep promotion efforts. Optimizing such interventions, particularly by addressing barriers to uptake, may strengthen their impact and broaden their applicability across inpatient settings.

## CONFLICT OF INTEREST STATEMENT

The authors declare no conflict of interest.

## ETHICS STATEMENT

This study was registered on ClinicalTrials.gov under trial number NCT04151251 prior to participant enrollment (https://clinicaltrials.gov/study/NCT04151251?a=9). This study was approved by the University of Chicago Institutional Review Board (protocol number 19‐0169). All participants provided written informed consent prior to their inclusion in the study. The study adhered to the Consolidated Standards of Reporting Trials (CONSORT) guidelines for reporting randomized controlled trials.

## Supporting information

Sunderrajan_ISLEEP Video.

## Data Availability

The individual de‐identified participant data (including the data dictionary) and all other materials are available from the corresponding author upon reasonable request. Data sharing is governed by institutional policies and follows the principles of confidentiality and participant protection.
